# Continuous monitoring of physiological data using the patient vital status fusion score in septic critical care patients

**DOI:** 10.1038/s41598-024-57712-9

**Published:** 2024-03-26

**Authors:** Philipp L. S. Ohland, Thomas Jack, Marcel Mast, Anette Melk, André Bleich, Steven R. Talbot

**Affiliations:** 1 Hannover Medical School, Institute for Laboratory Animal Science, Carl-Neuberg-Straße 1, 30625 Hannover, Germany; 2 Department of Pediatric Cardiology and Intensive Care Medicine, Hannover Medical School, Hanover, Germany; 3 Peter L. Reichertz Institute for Medical Informatics of TU Braunschweig and Hannover Medical School, Hanover, Germany; 4 Department of Pediatric Kidney, Liver and Metabolic Diseases, Hannover Medical School, Hanover, Germany

**Keywords:** Patient vital status, Quantitative scoring, Intensive care unit, Disease severity, Patient monitoring, Patient analysis tool, Data processing, Paediatric research

## Abstract

Accurate and standardized methods for assessing the vital status of patients are crucial for patient care and scientific research. This study introduces the Patient Vital Status (PVS), which quantifies and contextualizes a patient's physical status based on continuous variables such as vital signs and deviations from age-dependent normative values. The vital signs, heart rate, oxygen saturation, respiratory rate, mean arterial blood pressure, and temperature were selected as input to the PVS pipeline. The method was applied to 70 pediatric patients in the intensive care unit (ICU), and its efficacy was evaluated by matching high values with septic events at different time points in patient care. Septic events included systemic inflammatory response syndrome (SIRS) and suspected or proven sepsis. The comparison of maximum PVS values between the presence and absence of a septic event showed significant differences (SIRS/No SIRS: *p* < 0.0001, η^2^ = 0.54; Suspected Sepsis/No Suspected Sepsis: *p* = 0.00047, η^2^ = 0.43; Proven Sepsis/No Proven Sepsis: *p* = 0.0055, η^2^ = 0.34). A further comparison between the most severe PVS in septic patients with the PVS at ICU discharge showed even higher effect sizes (SIRS: *p* < 0.0001, η^2^ = 0.8; Suspected Sepsis: *p* < 0.0001, η^2^ = 0.8; Proven Sepsis: *p* = 0.002, η^2^ = 0.84). The PVS is emerging as a data-driven tool with the potential to assess a patient's vital status in the ICU objectively. Despite real-world data challenges and potential annotation biases, it shows promise for monitoring disease progression and treatment responses. Its adaptability to different disease markers and reliance on age-dependent reference values further broaden its application possibilities. Real-time implementation of PVS in personalized patient monitoring may be a promising way to improve critical care. However, PVS requires further research and external validation to realize its true potential.

## Introduction

In a fast-paced critical care environment, it is essential but challenging to accurately assess the severity of illness and the patient's overall condition^1,2^. This study proposes a novel approach that combines critical care-related variables with predetermined reference values, transforming continuously monitored vital signs into a time-sensitive, quantitative Patient Vital Status (PVS).

Despite advances in diagnostics and computation in the intensive care unit (ICU), traditional indicators such as skin complexion and capillary refill time remain invaluable^3^. However, these approaches may not be easily quantified or adapted to capture the many nuances of changes in patient status at every possible time point.

The challenges of deciphering complex patient data, big data analytics, and digital health applications are recognized as core components of critical care^4,5^. Clinical scores, e.g., determining the severity of illness, complement these efforts by consolidating and aggregating patient data^2,6^. Together, they can generate new knowledge, improve clinical practice, and advance healthcare by providing deeper insights and information on treatment decisions^7–10^.

Recent animal research has identified several characteristics contributing to the disease severity of animals undergoing experimental procedures in biomedical research^11–14^. By employing machine learning techniques to combine these multiple variables, it is possible to achieve improved validation and comprehensive understanding of severity classes compared to relying on single variables that may have high variance^15–17^.

The Relative Severity Assessment (RELSA) score represents the most recent and advanced method for integrative and multimodal quantitative severity assessment in preclinical settings. This validated approach calculates a latent severity scalar by comparing observed differences in outcome measures to a standardized reference set^18^. However, assessing disease severity in humans is more complex than in animals.

Systemic inflammatory response syndrome (SIRS) and sepsis in pediatric intensive care units (PICUs) and ICUs are common reasons for admission or occur during the stay^19,20^. Therefore, using individualized severity scores is critical to ensure qualitative assessments^21,22^. Timely detection and treatment are essential but challenging due to time constraints, limited resources, and complex diagnoses^23–26^. Sepsis-related scores, such as the Sepsis-related Organ Failure Assessment (SOFA) score, play a significant role in timely detection and intervention^27,28^. Other scores, such as the Sepsis Mortality Risk Score (SMRS), directly address sepsis patients and allow classification according to mortality risk^29–33^.

In the current landscape of critical care, assessing disease severity and detecting minor trend-indicating vital changes, especially for ICU patients, remains a complex challenge^10,34–37^. This requirement raises a crucial question: How can computational and data-driven methods be used to address this problem? Our study presents the Patient Vital Status, an adaptation of the RELSA method suited for human application. Using multiple clinical input variables provides a solution to minimize information loss, allows the comparison of individuals and groups, and generates disease-differentiating results using a fusion score^38^. It detects deviations from normative vital signs, most often associated with increasing disease severity.

In our application, we address the challenge of quantifying the vital status of patients with SIRS, suspected and proven sepsis in a PICU using retrospective, cross-sectional data. In this context, we hypothesize that the PVS can identify differences between patients with and without SIRS and those with more severe conditions such as sepsis.

## Material and methods

### PVS data collation and annotation

The data for this study were sourced from an existing data set of the PICU at the Hannover Medical School (MHH)^39^. The data underwent annotation and compilation, resulting in a finalized collection of tables. The selection criteria involved considering the measurement frequency, presence of vital parameters, and information about septic conditions. Any duplicate entries were removed. To calculate the composite PVS, only records containing values for all variables were utilized to guarantee complete data.

Seven outcome measures were utilized for the calculation: heart rate, peripheral pulse, respiratory rate, oxygen saturation, systolic blood pressure, mean arterial pressure, and temperature. The data included information about admission and discharge dates, date of birth, the time point of each measurement, and a unique but anonymized patient ID.

A moving average with a window size of five consecutive entries was calculated to mitigate potential noise in the raw data. This window size was chosen to emphasize the underlying trend and minimize the influence of single spurious peaks (Supplemental Material 1). For example, a smaller window size allowed an erroneously assessed 14% oxygen saturation to impact the saturation trend substantially. Due to the extensive data, the selected window size allowed us to filter out false data sufficiently. In addition, only temperatures above 34 °C were included to avoid biased results, e.g., by cooling mats. The data encompassed information regarding the timing of SIRS, proven sepsis, and suspected sepsis occurrences. The labeling followed the criteria reported at the 2005 International Pediatric Sepsis Consensus Conference^40^. In addition, the exact times these conditions occurred were utilized in calculating PVS changes. A patient could have more than one of these conditions. Individual time series data were merged on the time scale for continuous outcomes. Therefore, periods between measurements were not shown, and possible disease events and annotations were indexed on an absolute scale.

The final data contained 70 unique patients: 33 females (47%) and 37 males (53%) aged less than one year. Twenty-nine patients had no SIRS (41%), and 41 had SIRS (59%). Within this cohort, seven patients had proven sepsis, and 28 had suspected sepsis. No patient died.

### Establishment of the vital sign reference values

The reference values for heart rate, blood pressure, and respiratory rate used in this study, which represented the typical values for specific ages, were sourced from the European Resuscitation Council (ERC) Guidelines 2021 in Pediatric Life Support^41^. The midpoint between the upper and lower limits was used in the subsequent calculations. The fiftieth percentile was deployed for systolic blood pressure and mean arterial pressure (MAP). Reference values for oxygen saturation and body temperature were derived from other studies (Table 1)^42,43^.

**Table 1:** Age-dependent vital sign reference values.

Age (years)	Heart Rate (bpm)	Pulse (bpm)	Oxygen Saturation (%)	Respiratory Rate (rpm)	Systolic Blood Pressure (mmHg)	MAP (mmHg)	Temperature (°C)
0.08	145	145	100	42.50	75.00	55.00	37.30
1.00	135	135	100	35.00	95.00	70.00	37.30
2.00	125	125	100	29.00	96.25	71.25	37.30
5.00	105	105	100	23.50	100.00	75.00	37.30
10.00	90	90	100	19.50	110.00	75.00	37.30

### PVS calculation

The variables contributing to the fusion process resulting in the PVS were normalized according to the age-dependent reference values in Table 1 (Eq. 1). The patient's age was determined based on the date of birth and the measurement time. The reference age closest to the patient's age determined the respective reference values. The reference values did not change within one patient (e.g. because a different reference age became closer during the stay).

1 $$Value_{norm} = \frac{Value}{{Reference}}{*}100$$

Disease severity was associated with increasing or decreasing values. Therefore, deviations in either direction indicated aggravated conditions. For such ambivalent variables, the normalized values below 100% were corrected to an equivalent above 100% (Eq. 2).

2 $$Value_{norm} = 100 + \left( {100 - Value_{norm, < 100} } \right)$$

Since the basic functions of PVS are based on the RELSA procedure^18^, a reference was required that showed the maximum deviations in all contributing variables for a specific disease condition. Here, we specified the non-SIRS group as the reference. Consequently, the contrasts of the normalized values of each patient and the reference generated severity-related weights [w] for each variable. Finally, the combined weights were summarized with a root mean square procedure, resulting in a fused scalar called PVS. Thus, values above PVS = 1 indicated multidimensional measures that exceeded the highest severity in all contributing variables of the reference set.

### Statistical analyses

The PVS methodology was developed in R (v4.2.1). Data were processed using tidyverse, janitor, writexl, and readxl. Further, data were visualized with the ggplot2, ggsignif, and patchwork packages. The psych, lsr, lme4, lmerTest, car, zoo, rstatix, coin, and survival packages were used for statistical analyses.

The Shapiro–Wilk test was used to test against the hypothesis of normally distributed data. The Wilcoxon-Mann–Whitney test was utilized for non-normally distributed data. For normally distributed data with sample sizes of n = 30 and larger, two-tailed z-tests were used to compare the means of disease groups. Effect sizes were calculated as Cohen's d (for normally distributed data) or as Wilcoxon effect size (eta-squared (η^2^), for non-normally distributed data) to evaluate the potential relevance of clinical effects compared to inferential analyses dependent on sample sizes. To test bivariate correlations, we used the Pearson product-moment correlation coefficient. A generalized linear mixed-effects regression (GLMER) from the binomial family with anonymized patient IDs as random effects was used to test the individual vital signs as fixed effects on disease severity. The full model was tested against the null model with only the random effects in a likelihood ratio test to determine the effect of patient characteristics. Results of the GLMER were given as log-odds and the odds ratio (OR) with 95% confidence intervals. The vital sign data were normalized to zero mean and a standard deviation of 1 to ensure equal scales in the model. Trend analysis in single patient time series data was performed with a linear regression model, using the PVS as the dependent variable and time as the independent variable. The significance levels to interpret the resulting p-values were as follows: not significant (ns.), 0.05 (*), 0.01 (**), 0.001 (***), and 0.0001 (****).

### Ethical approval

All study participants, their parents, or legal guardians gave written informed consent. The study has been approved by the Ethics Committee of Hannover Medical School (No. 7804 BO S 2018 and No. 9819 BO S 2021). The study complied with the World Medical Association Declaration of Helsinki on Ethical Principles for Medical Research Involving Human Subjects and was reviewed by the Ethics Committee of Hannover Medical School. All methods were carried out in accordance with relevant guidelines and regulations.

## Results

### Cohort characterization

Table 2 characterizes the cohort, including information about the mean PICU stay and the average age of each group. Strikingly, the length of stay was significantly longer in patients with SIRS than those without (*p* < 0.0001, η^2^ = 0.53). In addition, the average length of stay was higher for proven sepsis (19.71 days) than for suspected sepsis (16.21 days).

**Table 2:** Cohort characterization and descriptive statistics.

	Total	Female	Male	No SIRS	SIRS	Suspected sepsis	Proven sepsis
n (%)	70 (100%)	33 (47%)	37 (53%)	29 (41%)	41 (59%)	28 (40%)	7 (10%)
Average stay (days)	11.30	13.03	9.43	6.14	14.66	16.21	19.71
Average age (days)	164.40	171.39	158.16	161.34	166.56	178.82	151.14
Minimal age (days)	32	32	37	32	37	37	44
Maximal age (days)	358	354	358	350	358	358	276

### Variable selection for PVS composite scoring

Pulse and systolic blood pressure were excluded from the PVS calculation because their severity weights were highly correlated with heart rate (ρ = 0.91, *p* < 0.0001) and MAP (ρ = 0.67, *p* < 0.0001). Therefore, the final variable set comprised heart rate, oxygen saturation, respiratory rate, MAP, and temperature, each showing low cross-correlation. For example, the highest correlation among the other variables was between heart rate and oxygen saturation with ρ = -0.24, which was considered acceptable for further analysis. All variables, except oxygen saturation, were considered ambivalent variables. Table 3 shows descriptive statistics of the final variable set. Supplemental Material 2 shows descriptive statistics divided into age groups of one month (n = 47) and one year (n = 23). Further, there was no significant difference between the sexes in the PVS, consistent with the reference values in the literature, which also reported no difference between sexes (Supplemental Material 3)^41^.

**Table 3:** Descriptive statistics of vital signs after applying rolling means.

	Heart rate (bpm)	Oxygen saturation (%)	Respiratory rate (rpm)	MAP (mmHg)	Temperature (°C)
Min	68.40	53.80	12.80	36.40	34.10
Max	213.20	100.00	112.20	135.60	40.80
Mean	123.60	95.67	35.00	57.92	37.02
Median	123.00	98.00	32.40	56.80	37.00

### Disease severity-related vital sign analysis in SIRS patients

A generalized linear mixed-effects regression model from the binomial family was used to evaluate the individual effects of each vital sign on the presence of SIRS, independent of time. The binary status of SIRS (present/not present) was the dependent variable, and the time factor was excluded from the analysis. Additionally, we accounted for the influence of patient IDs as random effects. We found the random effects component to have a significant variance (var = 9.16, Χ^2^ = 3829.8, df = 5, *p* < 0.0001), indicating that the patients' characteristics captured by the independent variables significantly influenced the outcome.

All vital signs were significant factors in the presence of SIRS (*p* < 0.0001). The log-odds of present SIRS increased with increasing oxygen saturation (0.36, OR = 1.43 CI_95%_[1.39; 1.49]), respiratory rate (0.27, OR = 1.31 CI_95%_[1.29; 1.33]), MAP (0.16, OR = 1.17 CI_95%_[1.16; 1.20]) and temperature (0.39, OR = 1.48 CI_95%_[1.45; 1.50]), whereas the log-odds decreased with increasing heart rate (-0.19, OR = 0.83 CI_95%_[0.81; 0.85]). The results showed that the included vital signs were differentially effective predictors in the presence of highly heterogeneous patient data (Supplemental Material 4).

### Using the PVS in continuous disease severity monitoring

**Figure 1:**
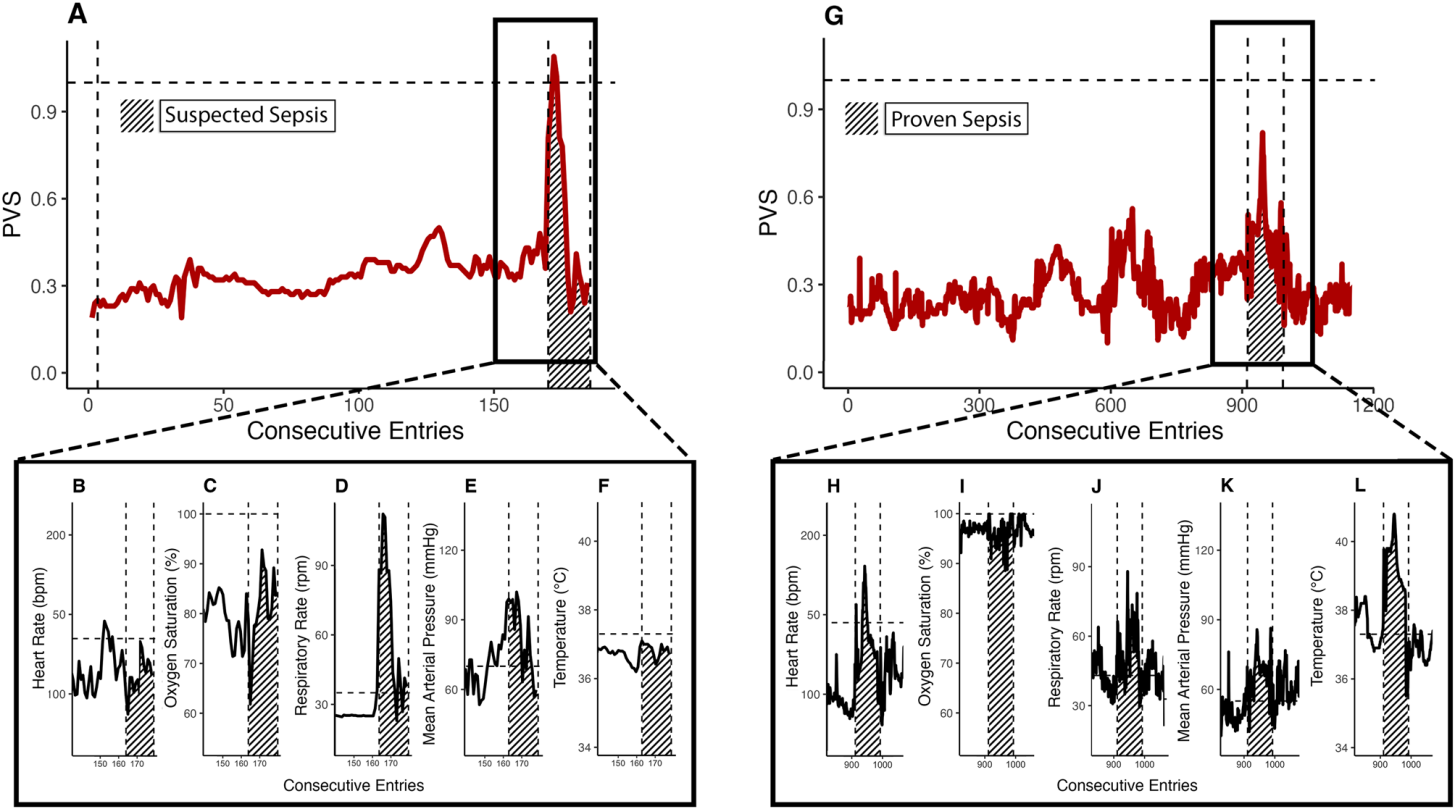
The patient vital status and corresponding vital signs during septic events. (**A**) The PVS development of the patient with the most severe PVS (PVS_max_ = 1.09) value. The PVS peak aligns with the independently annotated status of the suspected sepsis event (shaded area and vertically dashed lines), indicating a severe vital status during the septic state. The horizontally dashed line shows PVS = 1, the maximum PVS evaluated with baseline patients (non-SIRS patients). The vertical line indicates the end of a previously suspected sepsis before PICU admission. (**B**–**F)** Vital sign development during the suspected sepsis's PVS peak. Values range from the lowest to the highest possible value. The horizontally dashed line shows the reference level in each vital sign, indicating substantial deviations from expected normality. (**G)** The PVS development of the patient with the highest temperature (40.8 °C) and proven sepsis. Again, the highest PVS values match the proven septic event, indicating a severe vital status. (**H–L)** The vital sign contributions are different in this patient, with temperature and respiratory rate as the dominant variables. Notably, despite the temperature spikes, the PVS does not reach as high as the previous patient due to comparatively lower deviations in other vital signs. Data are shown as consecutive entries and not in real-time to avoid data gaps in the analysis.

The data showed that PICU patients exhibited substantial heterogeneity in values over time (Supplemental Materials 1 & 5). Therefore, as a use case, we focused on time series data from a patient with the highest PVS score (PVS = 1.09) to demonstrate disease monitoring. The vital sign values were also smoothed with a window size of 5 consecutive entries to exclude spurious spikes. In the following example, we will present the vital sign characteristics as the original measurement, the relative PVS weight (w), and the age-dependent reference values (ref) from the ERC Guidelines 2021 in Pediatric Life Support to provide situational context. The one-year-old male example patient had SIRS. The high PVS peak was consistent with co-occurring suspected sepsis (Fig. 1A). At the time of the highest PVS, the patient had a heart rate of 111 bpm (w = 0.37, ref = 135 bpm, 1B), oxygen saturation of 69.4% (w = 0.93, ref = 100%; 1C), respiratory rate of 112 rpm (w = 2.14, ref = 35 rpm; 1D), MAP of 98.8 mmHg (w = 0.59, ref = 70 mmHg; 1E), and temperature of 37 °C (w = 0.12, ref = 37.3 °C; 1F). The oxygen saturation and respiratory rate were mainly responsible for the PVS peak (Supplemental Material 5C-D). The average PVS for the entire PICU stay was PVS_av_ = 0.35, and the lowest PVS for this patient was 0.19.

The patient reached his lowest PVS on the first entry of his stay. He had the following vital signs at that time: a heart rate of 135 bpm (w = 0), oxygen saturation of 93.8% (w = 0.19), respiratory rate of 41.4 rpm (w = 0.18), MAP of 62.8 mmHg (w = 0.15), and a temperature of 38.1 °C (w = 0.31). As the temperature was slightly elevated, the temperature weight was mainly responsible for the elevated PVS. The difference between the last PVS before suspected sepsis (PVS = 0.36) and the maximum of the first four PVS values during suspected sepsis (PVS = 1.09) was ∆_PVS_ = 0.73. As the trend in this patient showed, the PVS peaked at the onset of suspected sepsis and decreased until the end of the suspected sepsis.

In contrast, a second male patient had SIRS but with proven sepsis. He was 0.37 years (4.4 months) old at admission. Although the patient had proven sepsis, the maximum PVS (0.82) was lower than in the first use case (Fig. 1G). However, he exhibited a spike in temperature (highest temperature in the data set) that was not present in the patient with suspected sepsis. Further, during the period of proven sepsis, the patient also had an elevated respiratory rate and MAP. The heart rate also showed a spike, while the oxygen saturation decreased (Fig. 1 H–L). The average PVS of this patient was PVS_av_ = 0.28, and the lowest possible PVS was 0.1 (Supplemental Material 6). The PVS values during proven sepsis (median = 0.47, IQR = 0.11) were significantly higher than those during times of no sepsis, showing a substantial effect (median = 0.25, IQR = 0.11); W = 5102, *p* < 0.0001, η^2^ = 0.4).

In both patients, slight trends before the sepsis events were visible in the time series but were difficult to observe when considering multiple vital parameters simultaneously. The fused PVS signal uncovered these trends and indicated a rising risk of health impairment through linear analysis over the entire observation time, culminating in the highest PVS peak (Patient 1: slope = 0.0055, df = 164, *p* < 0.0001; Patient 2: slope = 0.00064, df = 943, *p* < 0.0001).

### Larger PVS values corresponded with higher disease severity compared to values at patient discharge

The two use cases showed that suspected and actual sepsis events correlated with higher PVS values. However, in the current cross-sectional data, no controls were available. Therefore, we compared the maximum PVS [PVS_max_] in each group (No SIRS/SIRS, No Suspected Sepsis/Suspected Sepsis, and No Proven Sepsis/Proven Sepsis) with the last available PVS before discharge, assuming a lower disease-related burden for these values since no patient died. Also, the reason for the PICU stay was no longer present. Patients could have multiple status labels, i.e., some patients with suspected sepsis were also included in the group without proven sepsis, etc.

**Figure 2:**
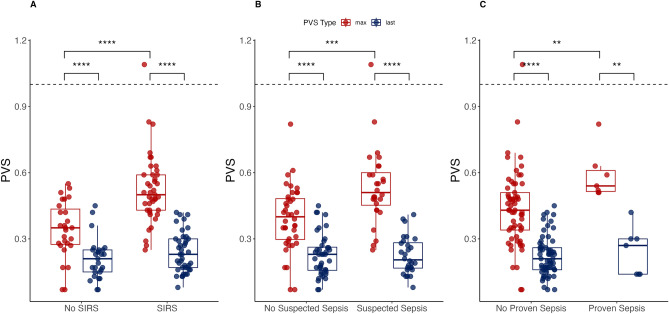
Maximum PVS values compared to higher disease severities and PVS values at discharge. (**A**) The patient cohort is divided into two groups (No SIRS/SIRS). The maximum PVS (PVS_max_; red) and the final PVS value before discharge from the PICU (PVS_last_; blue) are presented. Each patient is depicted as a dot, with the dotted line at PVS = 1 indicating the highest PVS observed in the reference patient group (i.e., non-SIRS patients). The difference in PVS_max_ between the two groups is statistically significant (*p* < 0.0001). Furthermore, the differences between PVS_max_ and PVS_last_ in both groups are also statistically significant (*p* < 0.0001), demonstrating the ability of PVS to distinguish between disease severities. It also suggests that these differences in severity are quantifiable when a patient with SIRS is followed over time and compared to patients without SIRS. (**B**) The patient cohort is divided into two groups based on suspected sepsis status: No Suspected Sepsis and Suspected Sepsis. PVS_max_ and PVS_last_ values are obtained from both groups. The difference in PVS_max_ between the two groups demonstrates a significant result (*p* = 0.00047), as the differences between PVS_max_ and PVS_last_ within each group (both *p* < 0.0001) do. (**C**) Based on proven sepsis status, the patient cohort is divided into two groups: No Proven Sepsis and Proven Sepsis, and PVS_max_ and PVS_last_ values are again presented. The difference in PVS_max_ between the two groups is significant (*p* = 0.0055), as well as the differences between PVS_max_ and PVS_last_ within each group (Proven Sepsis: *p* = 0.002; No Proven Sepsis: *p* < 0.0001). Notably, with a sample size of n = 7 proven septic patients, complete discrimination between PVS_max_ and PVS_last_ was observed.

All disease-related groups showed significantly higher PVS_max_ values than before discharge (Fig. 2). The effect sizes were considerably large (SIRS: *p* < 0.0001, η^2^ = 0.8; Suspected Sepsis: *p* < 0.0001, η^2^ = 0.8; Proven Sepsis: *p* = 0.002, η^2^ = 0.84). The corresponding groups without these conditions also showed significantly higher PVS_max_ values than before discharge: (No SIRS: *p* < 0.0001, η^2^ = 0.55; No Suspected Sepsis: *p* < 0.0001, η^2^ = 0.59; No Proven Sepsis: *p* < 0.0001, η^2^ = 0.66). Furthermore, the PVS_max_ values of disease-related groups were significantly higher than those of the groups without the corresponding disease (SIRS/No SIRS: *p* < 0.0001, η^2^ = 0.54; Suspected Sepsis/No Suspected Sepsis: *p* = 0.00047, η^2^ = 0.43; Proven Sepsis/No Proven Sepsis: *p* = 0.0055, η^2^ = 0.34). No significant differences were observed between the last PVS values in each condition, corroborating the assumption of stable patients immediately before discharge.

### Multidimensional PVS-related septic states were different from non-SIRS patients

**Figure 3:**
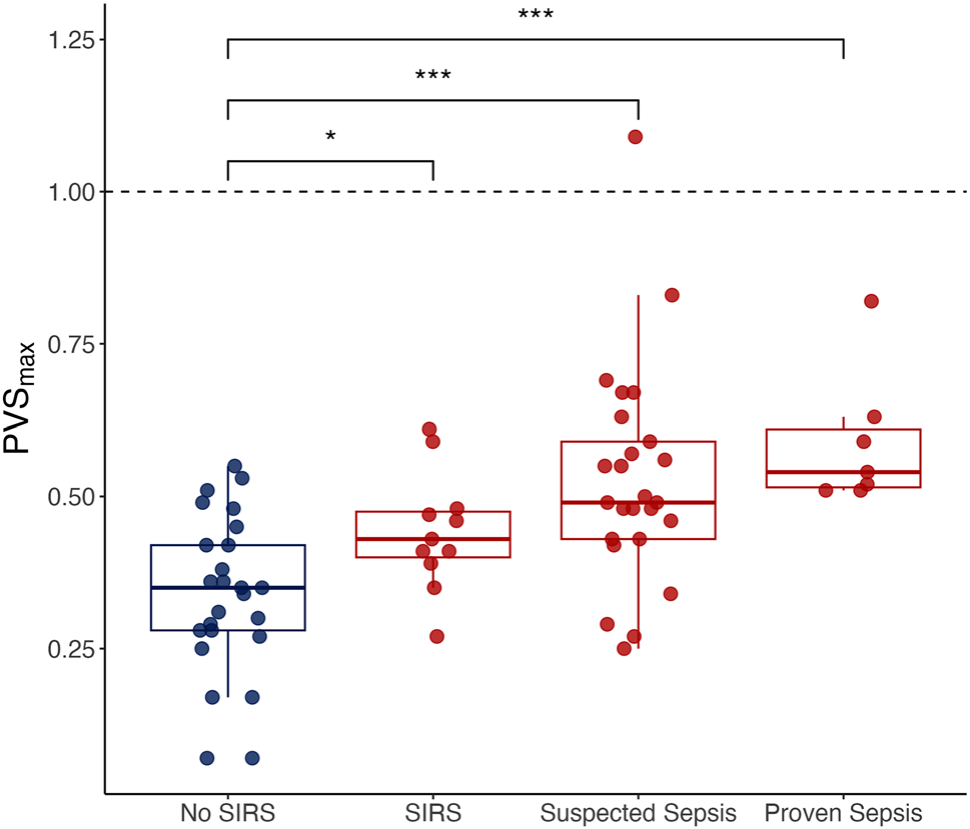
Discriminating different sepsis-states with the maximum PVS. With PVS_max_ on the y-axis and PVS = 1 as the reference maximum (non-SIRS patients) represented by a dotted horizontal line, the patient cohort is divided into four groups: No SIRS, SIRS, Suspected Sepsis, and Proven Sepsis. To prevent patient duplication across multiple groups in this specific analysis, they were placed in their highest disease group (the SIRS group includes only patients without suspected or proven sepsis). Therefore, each dot on the graph represents a unique patient. Each group was compared to the non-SIRS group, and significant differences were found (No SIRS/SIRS: *p* = 0.036; No SIRS/Suspected Sepsis: *p* = 0.00017; No SIRS/Proven Sepsis: *p* = 0.00031).

We analyzed the PVS_max_ values in four groups (No SIRS, SIRS (without suspected or proven sepsis), Suspected Sepsis, and Proven Sepsis) to compare septic states with non-SIRS patients. In the PICU setting, non-SIRS patients were still health-impaired, also showing elevated PVS values. However, three patients had both suspected and proven sepsis. Thus, they were categorized as having proven sepsis. Significant differences were observed between patients without and with SIRS (*p* = 0.036, η^2^ = 0.352) and patients without SIRS and suspected sepsis (*p* = 0.00017, η^2^ = 0.53). Finally, there was a significant difference in PVS_max_ values between non-SIRS patients and patients with proven sepsis (*p* = 0.00031, η^2^ = 0.64) (Fig. 3). Therefore, each additional disease severity category showed higher average PVS_max_ values than non-SIRS patients. However, the variance between septic states was considerable, which rendered SIRS-sepsis discrimination solely based on the PVS difficult without further information.

## Discussion

### PVS for continuous monitoring in patient care and research

There is a growing demand for more objective, comparable, and evidence-based methods to assess the quantified vital status of patients^4,7,8^. These methods are critical for improving the overall quality of research by providing higher standards for hypothesis testing, real-time patient monitoring, and data quality. In response to this need, the PVS was developed to address these issues by incorporating multiple outcome measures to form a composite and weighed score representing the body's current state. To indicate whether vital signs deviate from their normative values, the PVS focuses on out-of-norm conditions and changes in vital status. Therefore, we hypothesize that PVS identifies differences between patients with and without SIRS and those with more severe conditions such as sepsis. The basic idea for this approach is derived from the RELSA method^18^, which is successfully implemented in animal-based research to evaluate the severity of experimental procedures quantitatively^44–46^.

We adapted and translated the RELSA methodology to the clinical scale in this proof-of-concept study. Then, we retrospectively analyzed a cohort of PICU data obtained from very young patients aged 0 to 1 year. The corresponding analyses were challenging because of large variances, age-dependent baseline values, and the ambiguity of annotations. For example, we encountered spurious spikes in the data that had to be eliminated with a moving average to avoid false positives. Also, we found several unrealistic values (e.g., zero heart rate) even though the data were clinically validated. These examples illustrate that translating concepts from highly standardized animal experiments into human medicine can be difficult because real-world data introduces additional complexity.

Disease-related variable analysis revealed that the vital signs sometimes appeared to behave counterintuitively, e.g., the log-odds of present SIRS increased with increasing oxygen saturation. This demonstrated the complexity of the real-world data and may have been due to severe non-SIRS conditions, vital sign abnormalities that occurred only briefly during the SIRS period, and SIRS conditions with no abnormalities in one or more vital signs.

Nevertheless, valuable insights into the application of the PVS score were gained. We found, e.g., that all septic label types corresponded to higher maximum PVS scores (Fig. 2). Further, PVS scores also increased with a septic event and were significantly higher in the presence of proven sepsis. This finding corresponds with the result that the last PVS before discharge was significantly lower than the patients' maximum PVS, corroborating the research hypothesis of this study. These results imply that the PVS methodology provides a quantitative framework for continuously monitoring the vital status of patients.

This technology can be used to monitor disease progression and responses to treatment. Further, the PVS provides an overview of multidimensional patient data by reducing multiple occurring changes into one number. Theoretically, ICUs, medical facilities, and physicians can monitor many patients and conditions in parallel, while the PVS provides valuable input in decision-making processes during treatment. However, further studies are required to validate the procedure, e.g., for specific diseases, and compare the PVS with a defined set of variables to existing validated scores that use a similar methodology, such as the Pediatric Early Warning System (PEWS)^23,47^.

### Enhancing the precision for PVS assessment

We used a set of regularly monitored physical measures aligned with German ICU standards. The selection focused on continuously measured variables in this proof-of-concept, but other variables such as hemoglobin, C-reactive protein, or other clinical variables are also promising candidates^28,48,49^. We see this as an advantage of the PVS because it can be customized to particular disease markers while monitoring the overall medical situation. Further, physiological context is always maintained by referencing the measurements to reference values. The included variables did not show significant correlations, resulting in a diverse set of outcome measures that did not disproportionately influence the PVS calculation. Also, these variables had similar measurement frequencies to ensure the comparability and completeness of the results. Time-delayed measurements require data integration on larger time frames. While possible, this procedure always results in information loss and the necessity to impute data on a larger scale, which can be critical in medical decision-making processes.

The PVS generally remained low when variables evolved around the reference values. We visualized how the different variables affected the PVS in the presented use cases for suspected and proven sepsis (Fig. 1). The more variables deviated from the norm, the higher the PVS increased. Single outliers affected the PVS, but since the score is a weighted construct of multiple variables, substantial increases occurred only when multiple variables showed deviations from physiological levels. As in the RELSA, the root mean square calculation ensures that small fluctuations receive less weight in the final score, which helps minimize noise. However, future validation studies may require sensitivity analyses to address the problem of overfitting and construct validity for more specific disease-related questions.

Another critical aspect lies within the definition of reference values. Here, we included age-dependent values that changed considerably in the evaluated range from 0 to 1 year (Table 1). These references exhibit potential for improvement, e.g., smaller age scales offer more precise calculations of the PVS in pediatric patients when available. Adaptive reference values provide many possibilities for further customization, such as sex or specific medical conditions. Such adaptations could include, e.g., a fluid transformation of the oxygen saturation reference for patients with underlying cyanotic heart disease. We found no significant differences between the sexes due to the young patients’ ages. However, sex-specific effects can be expected in adults due to differences in reference values and the severity of illness in ICU patients^50–52^.

To comprehensively assess a patient's overall vital status, it is advisable to employ general variables or variables encompassing multiple aspects of the body. For instance, despite its normality in most measurements, incorporating body temperature as a variable proves advantageous as it indicates that the disease has not yet exerted a pervasive impact on the entire body. This holistic approach facilitates a more precise evaluation of the overall effect on a patient's vital status. Conversely, only pertinent variables associated with that particular disease should be incorporated when investigating a specific condition.

### PVS validation

The PVS is a mathematical tool that can help clinicians and researchers in disease-related decision-making processes. As such, it enables the possible evaluation of different drugs, conditions, therapy options, or treatment modalities on the patient's vital status. It provides an effective way of identifying substantial vital sign deviations and emerging disease trends. However, it is not suited for predicting the likelihood of death or specific outcomes such as ICU discharge. Instead, we focused on the patient's current state, enabling relative comparisons and differentiation between disease severities. A correlation was expected using similar variables as in the sepsis and SIRS definitions. However, the PVS has the potential to be extended to other clinically relevant variables.

In our example, SIRS or sepsis-related conditions showed spikes in the PVS. The maxima of these values (PVS_max_) were used to characterize and compare the maximum disease-related severity in multiple patients (Fig. 2). The comparisons showed significant differences in terms of the highest achieved severity. This outcome is relevant because sepsis or SIRS were only brief events during the entire PICU stay. Also, timely treatment may counteract the detection of escalating vital signs as values quickly return to lower numbers.

While we found significant differences between sepsis-related conditions, we also provided evidence-based effect sizes to emphasize the clinical potential of these findings. At the same time, the PVS_max_ comparisons showed large effect sizes between disease conditions, and the average PVS values of the entire data set showed no significant differences. This outcome was expected, as the lower PVS values in the time series diluted the spiking events.

Since the present data had no controls, we hypothesized that the PVS on the last day before discharge had better 'health quality' than during the PICU stay. When we compared these data to the PVS_max_ in the annotated regions of the disease-related data, we found significant differences in all groups concerning the maximum and discharge PVS values. In the case of proven sepsis, e.g., the vast effect size indicated PVS-related discrimination between septic patients and the respective data on discharge day (Fig. 2). Further, we compared the PVS values on discharge day between all conditions and found no significant difference. This analysis acted as internal control with externally annotated data and further confirmed the ability of the PVS to indicate deviations in the patients' vital status (Fig. 1). In addition, significant differences were observed when comparing non-SIRS patients with patients in any septic state, further emphasizing the potentially discriminative power of the score (Fig. 3).

### Limitations and prospects of PVS

The PVS is not a classic bedside score at this point, but it can be implemented in ICUs. It requires computational equipment, coding skills, and population-derived reference data. At this stage, the PVS differs from conventional scores used to assess, e.g., disease severity or risks. However, recent advances in artificial intelligence and other data-hungry processes have not stopped in medicine. They can potentially beat subjective scoring systems regarding precision, sensitivity, and specificity. Therefore, the PVS offers many options regarding future technological advances, such as disease and severity classification, risk assessment, and outcome prediction. However, in the current context of science and medicine, these methods serve only an advisory role. Despite remarkable advancements in the data sciences, the authority for interpretation and decision-making will remain in human hands for the foreseeable future.

Also, a more user-friendly application with less coding and better accessibility via a graphical user interface, such as a mobile phone application, is likely to enhance the acceptance and usability of the PVS method after future developments. Alternatively, the PVS could easily be implemented in any stationary ICU monitoring equipment. However, a deeper understanding and characterization of disease-specific variables and their sensitivity is needed for these prospects. Since any medicine-related measurements can be included in the PVS, new combinations, markers, and discriminators that accurately characterize specific diseases and the corresponding recovery processes may be found.

An essential aspect of the PVS calculation is the implemented scaling of the input variables before weight calculations. In the vital parameters, the measured ranges are different. While a temperature deviation of a few degrees is life-threatening, a higher heart rate, for example, is temporarily survivable. This range aspect is accurately addressed in the PVS score. Therefore, with sufficient data, it is possible to implement a risk prediction system using PVS scores in the future. However, while this study showed that the PVS could discriminate between the absence and presence of proven sepsis, it is essential to note that the score is not predictive using the data presented herein. The reason for this lies in the annotation of the data. Predictions require a model that is trained with labeled data. In the current data, patients were marked SIRS or suspected sepsis by the clinicians based on external parameters (see the Methods section) that were not optimized for machine learning.

Interestingly, many data points do not show any corresponding changes or noticeable deviations in vital signs. Without proper annotation or rules, the PVS introduces this annotation bias and further variance into any discriminatory system. Other methods must be found to discriminate between time series data and conditional transitions for specific diseases. This challenge also means that the PVS does not explain why a patient has a particular health status.

Furthermore, a physiological deviation of vital parameters also leads to a deviation in the PVS, with no underlying increase in disease severity. Still, the methodology can be adapted to specific clinical contexts that decide how to interpret future PVS applications. In this study, an increase in PVS was correlated with disease severity as physiological vital changes were not expected in a PICU setting with invasive blood pressure monitoring.

Future applications must address false values derived from, e.g., misplaced sensors and technical or human errors. It is also possible to use other artificial intelligence tools, such as the Random Forest model, to distinguish between accurate alerts and artifacts^53,54^.

The PVS aligns seamlessly with the computational methods prevalent in modern medical practice. In line with surgical robotics or fall detection devices, PVS supports assessing a patient's current vital status by fusing sensor data into one number – a technology that could easily be integrated into wearables, etc. Nevertheless, the PVS's potential and visionary application is its use in real-time vital status monitoring in ICUs and PICUs. This innovative application would provide invaluable information about improving or deteriorating patient conditions, treatment effects, and recovery. Ultimately, this enables healthcare professionals to respond more quickly to changes, which helps improve patients' health.

## Conclusion

Our study demonstrates the substantial potential of the PVS as a quantitative tool for assessing the continuous physical status in an ICU setting. Although the PVS faces challenges from biased annotations, variable selection, and variance, it is well-suited to monitor the multidimensional vital status of individual patients. Therefore, PVS can be envisioned as a real-time patient monitoring method in critical care. Its potential ability to classify disease severity and monitor the interaction of different health markers provides a rich scope for study. Within this application alone, the PVS offers outstanding potential to improve patient care and adds meaningful contributions to critical care medicine. However, future research must explore its full potential, especially its broader applicability in other areas of medicine.

### Supplementary Information

Supplementary Information.

## Data Availability

The PVS code and documentation are available on GitHub (https://github.com/phohland/PVS). The data that support the findings of this study are available from^41^. Still, restrictions apply to the availability of these data, which were used under license for the current study and are not publicly available. Data are, however, available from the authors upon reasonable request and with permission of the ELISE project (https://www.zdin.de/digitales-niedersachsen/projektubersicht/elise).

## Abbreviations

ERC: European resuscitation council
ICU: Intensive care unit
MHH: Hannover medical school
MAP: Mean arterial pressure
PVS: Patient vital status
PEWS: Pediatric early warning system
PICU: Pediatric intensive care unit
RELSA: Relative severity assessment score
SMRS: Sepsis mortality risk score
SIRS: Systemic inflammatory response syndrome
SOFA: Sepsis-related organ failure assessment Score
bpm: Beats per minute
η^2^: Eta-squared
m: Number of entries
mmHg: Millimeter mercury
n: Sample size
PVS_max_: Maximum patient vital status
PVS_av_: Average patient vital status
ref: Reference value
ρ: Pearson's correlation coefficient
rpm: Respirations per minute
SD: Standard deviation
ID: Patient identification number
w: Severity weight
