# Supplementary material for: Continuous monitoring of physiological data using the patient vital status fusion score in septic critical care patients
Source: Sci Rep. 2024 Mar 26;14:7198. doi: 10.1038/s41598-024-57712-9 (PMC10965972; doi:10.1038/s41598-024-57712-9)
Supplement: Supplementary file 1 — Supplementary Information. [file 41598_2024_57712_MOESM1_ESM.docx]

Supplemental Material

# 1 Data Preparation

Patient raw data were smoothed with a rolling average using a window size of five consecutive entries. This procedure mitigated the potential influence of short-term artifacts and incorrect measurements that would otherwise result in false or inflated peaks in the data. Data in panel S1 are represented as consecutive entries.


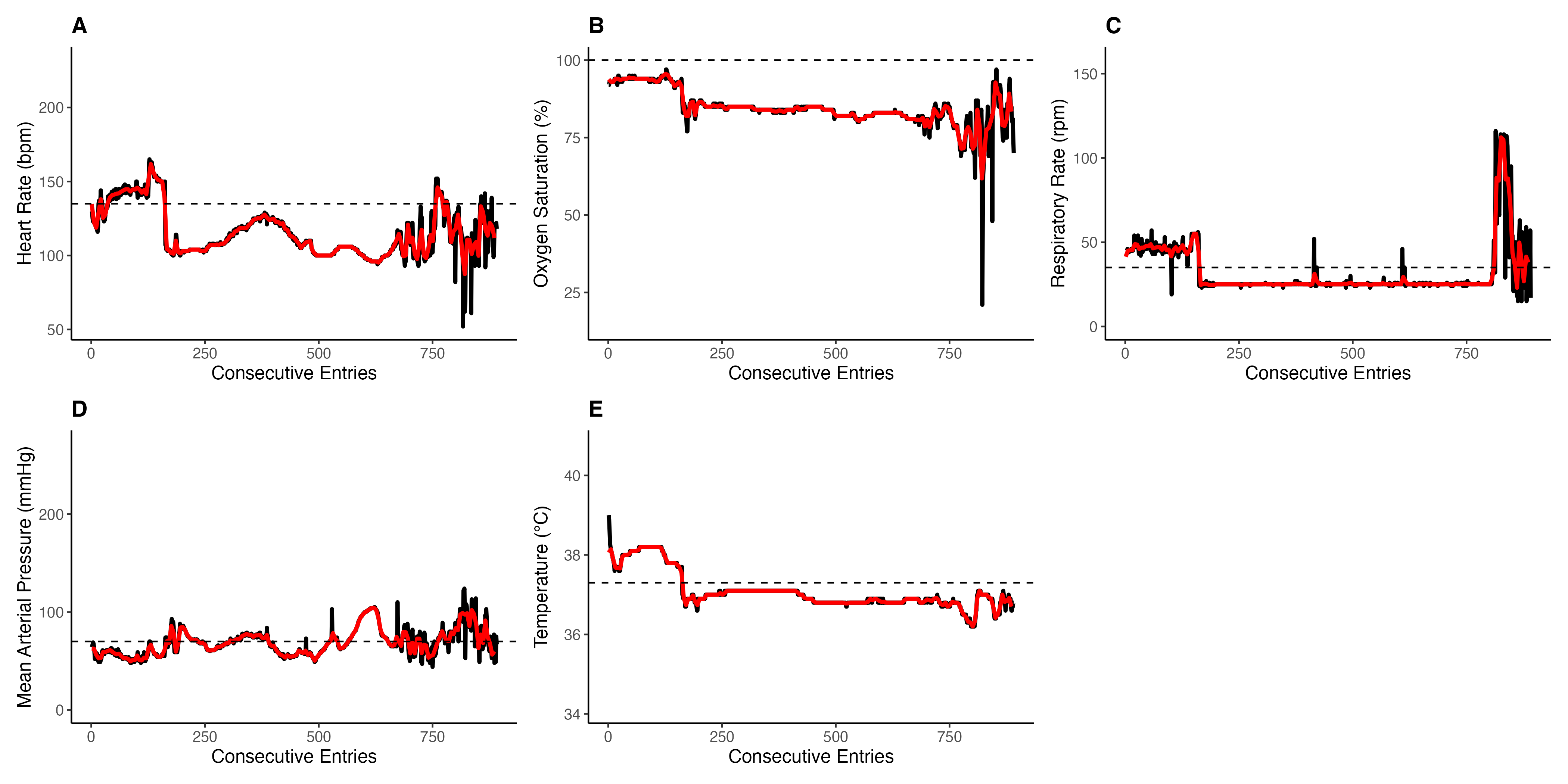


**Figure S1.** Raw (black) and smoothed (red) data from the patient with the highest PVS in the analyzed data. The used exogenic variables of the PVS are shown in (**A**) heart rate, (**B**) oxygen saturation, (**C**) respiratory rate, (**D**) mean arterial blood pressure, and (**E**) temperature. Note that several spikes in the data do not necessarily provide more information or show unrealistic values.

**2 Descriptive statistics of vital signs divided into age groups**

The patients were divided into age groups according to their nearest reference age: one month (0.08 years) and one year. The division does not consider potential differences in disease status between age groups.

**Table S1.** Descriptive statistics of vital signs after applying rolling means (age: one month).

|  | **Heart Rate (bpm)** | **Oxygen Saturation (%)** | **Respiratory Rate (rpm)** | **MAP (mmHg)** | **Temperature (°C)** |
| --- | --- | --- | --- | --- | --- |
| **Min** | 70.80 | 53.80 | 12.80 | 36.40 | 34.10 |
| **Max** | 199.00 | 100.00 | 87.4 | 108.20 | 40.80 |
| **Mean** | 132.00 | 93.90 | 33.00 | 56.28 | 36.98 |
| **Median** | 133.4 | 96.00 | 31.40 | 55.00 | 36.90 |

**Table S2.** Descriptive statistics of vital signs after applying rolling means (age: one year).

|  | **Heart Rate (bpm)** | **Oxygen Saturation (%)** | **Respiratory Rate (rpm)** | **MAP (mmHg)** | **Temperature (°C)** |
| --- | --- | --- | --- | --- | --- |
| **Min** | 68.40 | 61.80 | 14.80 | 39.2 | 34.12 |
| **Max** | 213.20 | 100.00 | 112.20 | 135.60 | 38.80 |
| **Mean** | 114.20 | 97.64 | 37.22 | 59.74 | 37.07 |
| **Median** | 112.40 | 99.80 | 34.60 | 58.60 | 37.08 |

# 3 Between sex comparison of PVS_max_

**
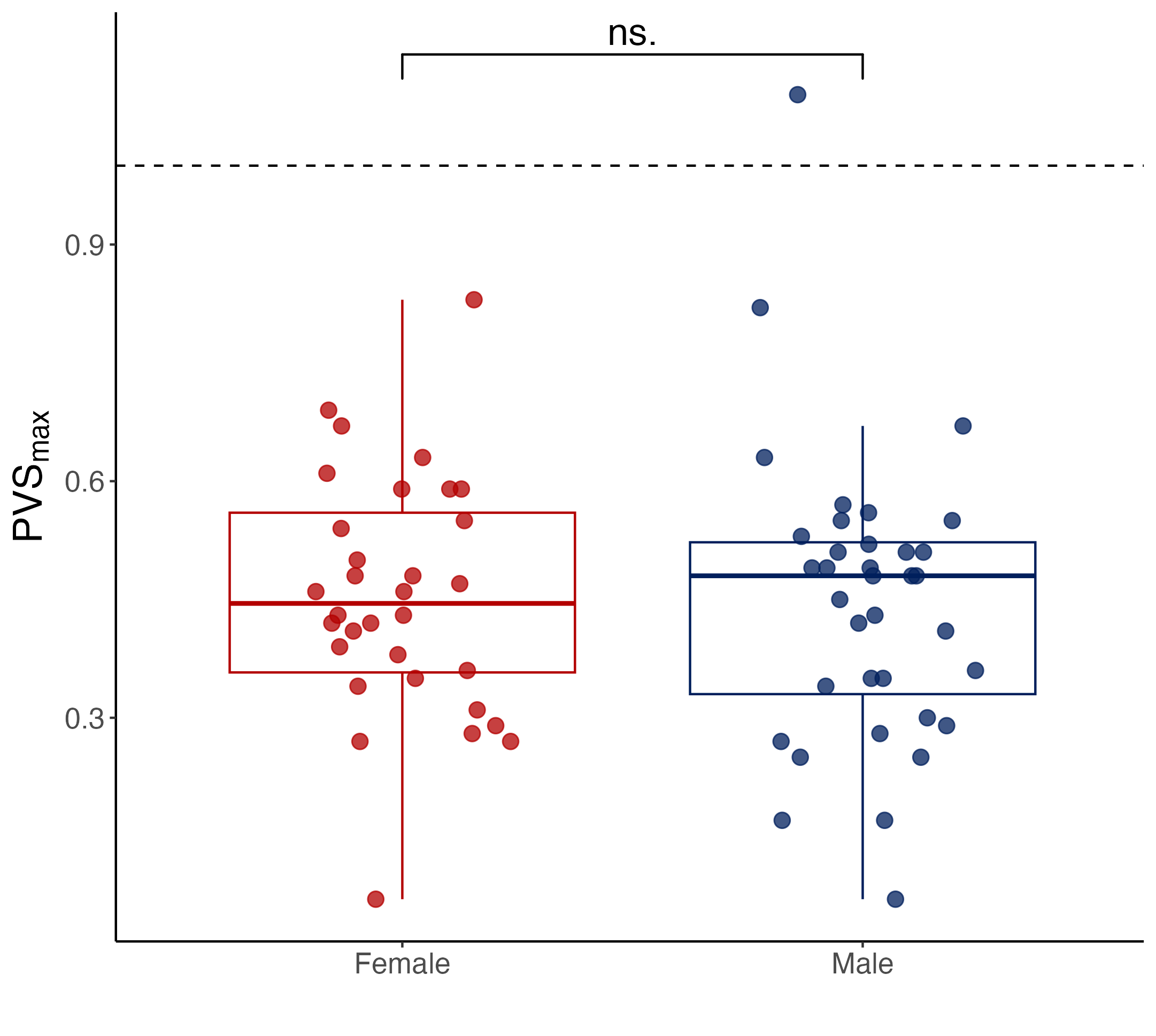
**

**Figure S2.** Each point represents the PVS_max_ value of a patient. Data were tested against the hypothesis of normal distribution with the Shapiro-Wilk test. In the male patients, the normal distribution could not be assumed (p < 0.05). Therefore, robust pairwise analysis was conducted with the non-parametric Wilcoxon-Mann-Whitney test. Female patients (median = 0.44, IQR = 0.2; red) did not significantly differ from male patients (median = 0.48, IQR = 0.19; blue) in terms of PVS_max_ (W = 598, p = 0.79, η^2^ = 0.03).

# 4 Disease severity-related vital sign analysis in SIRS patients

The severity-related vital signs of putative SIRS patients were tested with a generalized linear mixed-effects regression of the binominal family. Data were annotated by clinicians depending on the individual patient status. Here, the quality of SIRS was defined by presence or absence at any given time point. Due to the particular time series in each patient, we set the patient IDs as random effects to account for individual variance. Further, in this more general approach, time was excluded as a factor. Before the analysis, all independent variables were scaled and centered to adjust for the different scales (z-score transformation). We used the following (full) model to obtain the estimates of the SIRS status.

*SIRS ~ heart rate (scaled) + oxygen saturation (scaled) + respiratory rate (scaled) + MAP (scaled) + temperature (scaled) + (1 | patient ID)*

Table S3 shows the coefficients of the resulting model. The random effect variance (intercept) among patients was 9.16, suggesting patient-specific differences. Comparing the full model to the null model with only the random intercept term showed the significant influence of individual patient characteristics on the model (Χ^2^=3829.8, df=5, p < 0.0001). The random intercept term in the full model ensures that these characteristics are included in the estimate approximations. In total, 131148 observations were recorded across 70 distinct patients

**Table S3.** Fixed effects of the generalized linear mixed-effects regression.

|  | **Estimate** | **Std. Error** | **z-value** | **p-value** |  |
| --- | --- | --- | --- | --- | --- |
| **(Intercept)** | -4.18 | 0.40 | -10.43 | < 0.0001 | **** |
| Heart Rate (scaled) | -0.19 | 0.01 | -16.47 | < 0.0001 | **** |
| Oxygen saturation (scaled) | 0.36 | 0.02 | 20.5 | < 0.0001 | **** |
| Respiratory Rate (scaled) | 0.27 | 0.01 | 33.76 | < 0.0001 | **** |
| MAP (scaled) | 0.16 | 0.01 | 19.23 | < 0.0001 | **** |
| Temperature (scaled) | 0.39 | 0.01 | 42.52 | < 0.0001 | **** |

**5 PVS and vital signs of the patient with the most severe PVS**


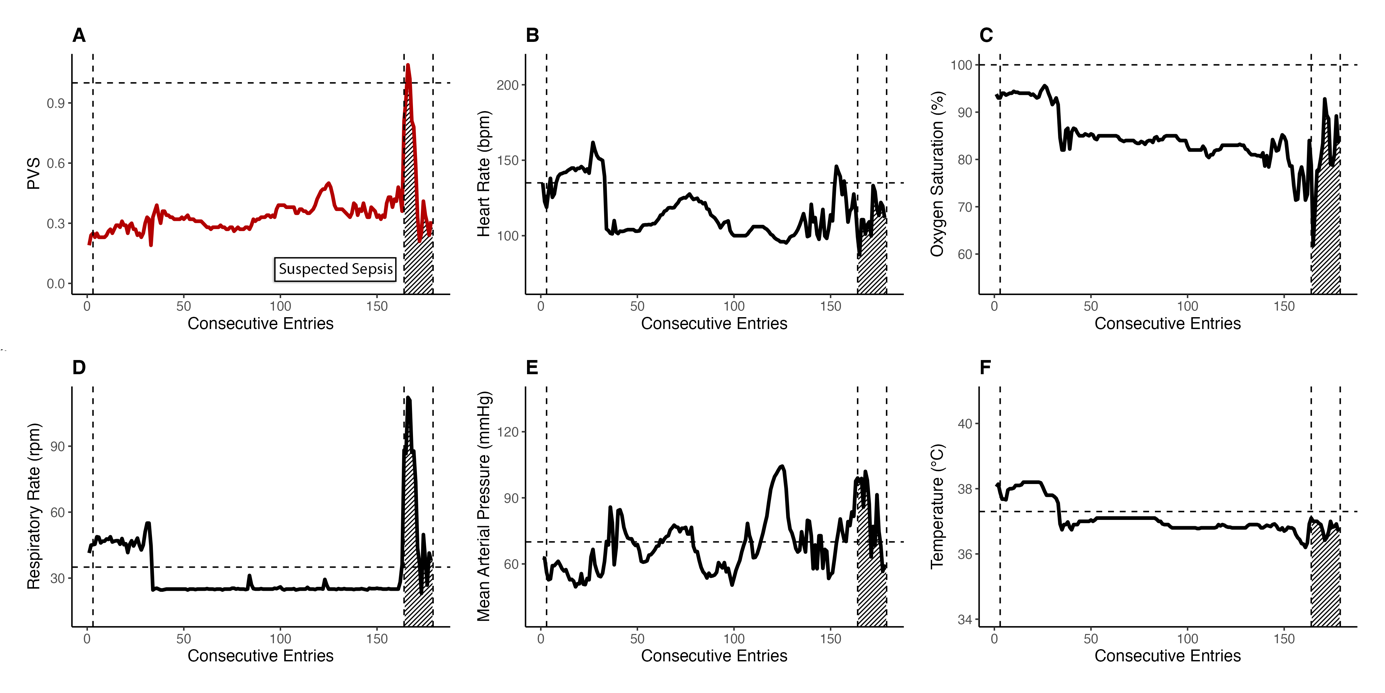


**Figure S4.** PVS and vital sign panel of the patient with the most severe PVS in the analyzed data (PVS_max_=1.09). (**A**) The combined PVS signal (red). The peak value corresponded precisely with the clinically annotated suspected sepsis event (shaded areas). The horizontal dotted line in the plot represents a PVS of 1, representing the maximum in all contributing vital signs of the reference set (here: the non-SIRS group). (**B-F**) The individual vital sign data contributing to the PVS are also labeled (shaded) for the sepsis event. The vertically dotted lines mark the beginning and the end of the labeled suspected sepsis phase. The vertically dotted lines at the beginning of plots **B-F** indicate the end of the suspected sepsis before the patient was admitted to the PICU.

**6 PVS and vital signs of the patient with the highest temperature**


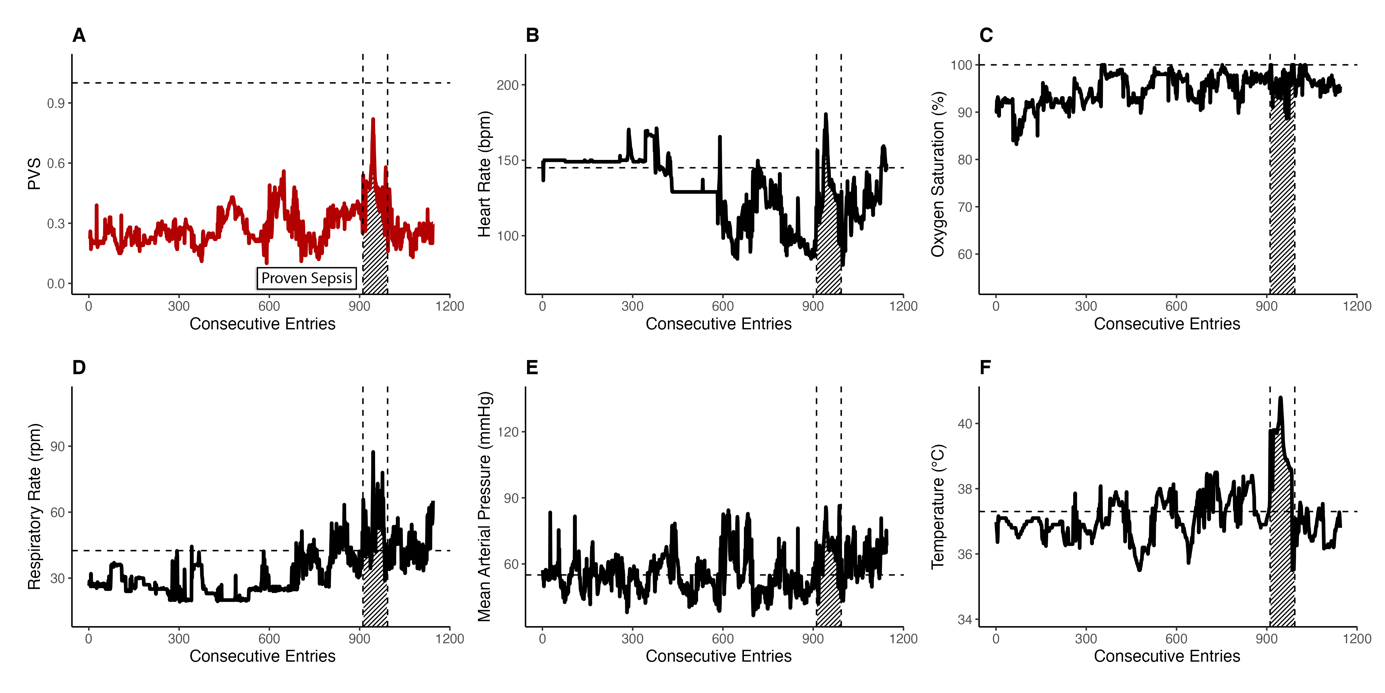


**Figure S5.** The patient's PVS and vital sign panel exhibited the highest recorded temperature within the analyzed dataset (temperature = 40.8°C). (**A**) The combined PVS signal (red). The most prominent peak aligns with the clinically annotated period of proven sepsis (shaded areas). Before the onset of sepsis, the patient had experienced smaller peaks. The horizontal dashed line on the graph represents a PVS value of 1. (**B-F**) The individual vital sign data contributing to the PVS are also indicated (shaded) for the septic event. Vertical dashed lines demarcate the beginning and ending of the proven sepsis phase. Of particular note is the precise peak observed in temperature, respiratory rate, and heart rate during the septic episode.
